# Methyl (9aR*,10S*,11R*,13aS*,13bS*)-9-oxo-6,7,9,9a,10,11-hexa­hydro-5*H*,13b*H*-11,13a-ep­oxy­pyrrolo­[2′,1′:3,4][1,4]diazepino[2,1-*a*]isoindole-10-carboxyl­ate

**DOI:** 10.1107/S1600536811040360

**Published:** 2011-10-05

**Authors:** Flavien A. A. Toze, Inga K. Airiyan, Eugeniya V. Nikitina, Elena A. Sorokina, Victor N. Khrustalev

**Affiliations:** a Department of Chemistry, University of Douala, Faculty of Sciences, PO Box 24157, Douala, Republic of Cameroon; b Department of Organic Chemistry, Russian Peoples’ Friendship University, 6 Miklukho-Maklaya St, Moscow 117198, Russian Federation; c X-Ray Structural Centre, A.N. Nesmeyanov Institute of Organoelement Compounds, Russian Academy of Sciences, 28 Vavilov St, B-334, Moscow 119991, Russian Federation

## Abstract

The title compound, C_17_H_18_N_2_O_4_, is the methyl ester of the adduct of intra­molecular Diels–Alder reaction between maleic anhydride and 1-(2-fur­yl)-2,3,4,5-tetra­hydro-1*H*-pyrrolo­[1,2-*a*][1,4]diazepine. The mol­ecule comprises a fused penta­cyclic system containing four five-membered rings (*viz*. pyrrole, 2-pyrrolidinone, tetra­hydro­furan and dihydro­furan) and one seven-membered ring (1,4-diazepane). The pyrrole ring is approximately planar (r.m.s. deviation = 0.003 Å) while the 2-pyrrolidinone, tetra­hydro­furan and dihydro­furan five-membered rings have the usual envelope conformations. The central seven-membered diazepane ring adopts a boat conformation. In the crystal, mol­ecules are bound by weak inter­molecular C—H⋯O hydrogen-bonding inter­actions into zigzag chains propagating in [010]. In the crystal packing, the chains are stacked along the *a* axis.

## Related literature

For reviews on the synthesis of isoindoles, see: Jones & Chapman (1996 ▶); Donohoe (2000 ▶). For reviews on intra­molecular cyclo­addition reactions of α,β-unsaturated acid anhydrides to furfuryl­amines (IMDAF reactions), see: Vogel *et al.* (1999 ▶); Zubkov *et al.* (2005 ▶). For related compounds, see: Zubkov *et al.* (2009 ▶, 2010 ▶, 2011 ▶).
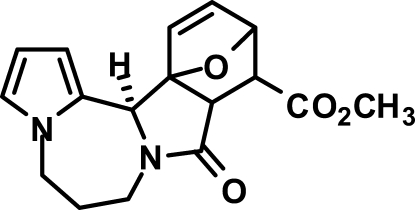


## Experimental

### 

#### Crystal data

C_17_H_18_N_2_O_4_
*M* *_r_* = 314.33
Monoclinic, 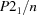

*a* = 11.5817 (12) Å
*b* = 9.0152 (10) Å
*c* = 14.8607 (16) Å
β = 112.749 (2)°
*V* = 1430.9 (3) Å^3^
*Z* = 4
Mo *K*α radiation
μ = 0.11 mm^−1^
*T* = 100 K
0.30 × 0.20 × 0.20 mm

#### Data collection

Bruker APEXII CCD diffractometer
Absorption correction: multi-scan (*SADABS*; Sheldrick, 2003 ▶) *T*_min_ = 0.969, *T*_max_ = 0.979
17904 measured reflections
4167 independent reflections
3685 reflections with *I* > 2σ(*I*)
*R*_int_ = 0.028

#### Refinement

*R*[*F* ^2^ > 2σ(*F* ^2^)] = 0.037
*wR*(*F* ^2^) = 0.099
*S* = 1.00
4167 reflections
209 parameters
H-atom parameters constrained
Δρ_max_ = 0.40 e Å^−3^
Δρ_min_ = −0.25 e Å^−3^

### 

Data collection: *APEX2* (Bruker, 2005 ▶); cell refinement: *SAINT-Plus* (Bruker, 2001 ▶); data reduction: *SAINT-Plus*; program(s) used to solve structure: *SHELXTL* (Sheldrick, 2008 ▶); program(s) used to refine structure: *SHELXTL*; molecular graphics: *SHELXTL*; software used to prepare material for publication: *SHELXTL*.

## Supplementary Material

Crystal structure: contains datablock(s) global, I. DOI: 10.1107/S1600536811040360/aa2027sup1.cif

Structure factors: contains datablock(s) I. DOI: 10.1107/S1600536811040360/aa2027Isup2.hkl

Additional supplementary materials:  crystallographic information; 3D view; checkCIF report

## Figures and Tables

**Table 1:** Hydrogen-bond geometry (Å, °)

*D*—H⋯*A*	*D*—H	H⋯*A*	*D*⋯*A*	*D*—H⋯*A*
C9*A*—H9*A*⋯O1^i^	1.00	2.43	3.4334 (13)	180

Symmetry code: (i) 
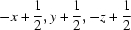
.

**Fig. 1.**
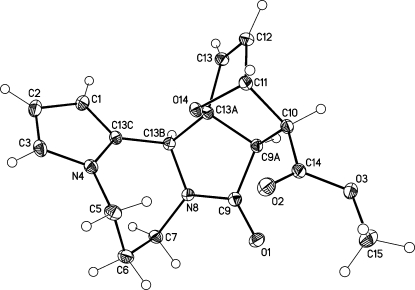
Crystal structure of (I). Displacement ellipsoids are shown at the 50% probability level.

**Fig. 2.**
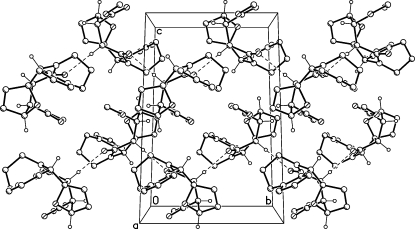
Crystal packing of (I) along the a axis. Dashed lines indicate the weak intermolecular C—H···O hydrogen bonding interactions.

**Fig. 3.**
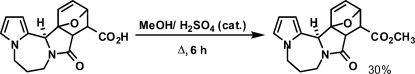
Esterification of 11,13a-epoxypyrrolo[2',1':3,4][1,4]diazepino[2,1-a]isoindole-10-carboxylic acid.

## supplementary crystallographic information

### Comment

In the last ten years our group have developed an effective strategy for the synthesis of isoindoles (Donohoe, 2000; Jones & Chapman, 1996) and 3,6a-epoxyisoindoles (Vogel *et al.*, 1999) annulated with various heterocycles (Zubkov *et al.*, 2009, 2010, 2011). This strategy was based on the intramolecular cycloaddition reaction of α,β-unsaturated acid anhydrides to furfurylamines (IMDAF) (Zubkov *et al.*, 2005).

This article describes the synthesis of a novel heterocyclic pyrrolo[2',1':3,4][1,4]diazepino[2,1-*a*]isoindole system, which can be easily obtained using the IMDAF reaction between maleic anhydride and 1-(2-furyl)-2,3,4,5-tetrahydro-1*H*-pyrrolo[1,2-*a*][1,4]diazepine (Zubkov *et al.*, 2011).

The molecule of compound (I), C_17_H_18_N_2_O_4_, comprises a fused pentacyclic system containing four five-membered rings (pyrrole, 2-pyrrolidinone, tetrahydrofuran and dihydrofuran) and one seven-membered ring (1,4-diazepane) (Figure 1). The pyrrole ring is planar, and the 2-pyrrolidinone, tetrahydrofuran and dihydrofuran five-membered rings have usual *envelope* conformations. The central seven-membered diazepane ring adopts a *boat* conformation. The nitrogen N4 atom has a trigonal-planar geometry (sum of the bond angles is 360.0°), whereas the nitrogen N8 atom is slightly pyramidalized (sum of the bond angles is 359.1°). The *boat* bottom of the diazepane ring (N4–C6–C7–C13C) is practically perpendicular to the base plane of the pyrrolidinone ring (N8–C9–C13A–C13B) (the dihedral angle is 85.52 (4)°).

The molecule of (I) possesses five asymmetric centers at the C9A, C10, C11, C13A and C13B carbon atoms and can have potentially numerous diastereomers. The crystal of (I) is racemic and consists of enantiomeric pairs with the following relative configuration of the centers: *rac*-9 A*R**,10*S**,11*R**,13 A*S**,13B*S**.

In the crystal, the molecules of (I) are bound by the weak intermolecular C–H···O hydrogen bonding interactions into the *zigzag*-like chains toward [010] (Figure 2, Table 1). The crystal packing of the chains is stacking along the *a* axis (Figure 2).

### Experimental

A solution of the acid (2.0 g, 6.7 mmol) in methanol (40 ml) was refluxed for 6 h in the presence of catalytic amount of concentrated H_2_SO_4_ (monitoring by TLC until disappearance of the starting compound sport, eluent – EtOAc, Sorbfil) (Figure 3). At the end of the reaction, the clear brown solution was poured into water (250 ml) and extracted with CHCl_3_ (3×70 ml). The extract was dried over MgSO_4_ and concentrated in *vacuo*. The crude ester was recrystallized from a mixture of PrOH–DMF to give the title compound as colourless prisms. Yield 30%. The single crystals of the product were obtained by slow crystallization from methanol (yield 52%). *M.p*.= 458–460 K. *R_f_* 0.51 (ethyl acetate, Sorbfil).

### Refinement

The hydrogen atoms were placed in calculated positions with C–H = 0.95–1.00 Å and refined in the riding model with fixed isotropic displacement parameters [*U*_iso_(H) = 1.5*U*_eq_(C) for CH_3_-groups and *U*_iso_(H) = 1.2*U*_eq_(C) for the other groups].

### Crystal data

**Table:**

C_17_H_18_N_2_O_4_	*F*(000) = 664
*M**_r_* = 314.33	*D*_x_ = 1.459 Mg m^−^^3^
Monoclinic, *P*2_1_/*n*	Mo *K*α radiation, λ = 0.71073 Å
Hall symbol: -P 2yn	Cell parameters from 7472 reflections
*a* = 11.5817 (12) Å	θ = 2.7–32.6°
*b* = 9.0152 (10) Å	µ = 0.11 mm^−^^1^
*c* = 14.8607 (16) Å	*T* = 100 K
β = 112.749 (2)°	Prism, colourless
*V* = 1430.9 (3) Å^3^	0.30 × 0.20 × 0.20 mm
*Z* = 4	

### Data collection

**Table:**

Bruker APEXII CCD diffractometer	4167 independent reflections
Radiation source: fine-focus sealed tube	3685 reflections with *I* > 2σ(*I*)
graphite	*R*_int_ = 0.028
φ and ω scans	θ_max_ = 30.0°, θ_min_ = 1.9°
Absorption correction: multi-scan (*SADABS*; Sheldrick, 2003)	*h* = −16→16
*T*_min_ = 0.969, *T*_max_ = 0.979	*k* = −12→12
17904 measured reflections	*l* = −20→20

### Refinement

**Table:**

Refinement on *F*^2^	Primary atom site location: structure-invariant direct methods
Least-squares matrix: full	Secondary atom site location: difference Fourier map
*R*[*F*^2^ > 2σ(*F*^2^)] = 0.037	Hydrogen site location: inferred from neighbouring sites
*wR*(*F*^2^) = 0.099	H-atom parameters constrained
*S* = 1.00	*w* = 1/[σ^2^(*F*_o_^2^) + (0.0545*P*)^2^ + 0.545*P*] where *P* = (*F*_o_^2^ + 2*F*_c_^2^)/3
4167 reflections	(Δ/σ)_max_ < 0.001
209 parameters	Δρ_max_ = 0.40 e Å^−^^3^
0 restraints	Δρ_min_ = −0.25 e Å^−^^3^

### Special details

**Table:**

Experimental. IR (KBr), ν (cm^-1^): 3406, 1737, 1690; ^1^H NMR (CDCl_3_, 600 MHz, 300 K): δ = 1.83 (m, 1H, H6A), 2.16 (m, 1H, H6B), 2.78 (d, 1H, H9A, J_9A,10_ = 8.9), 2.84 (ddd, 1H, H6, J_7B,6B_ = 6.9, J_7B,6A_ = 10.6, J_7,7_ = 17.9), 2.91 (d, 1H, H10, J_9A,10_ = 8.9), 3.79 (s, 3H, CO_2_*Me*), 3.81 (ddd, 1H, H5B, J_5B,6A_ = 5.8, J_5B,6B_ = 8.3, J_5,5_ = 14.2), 4.02 (br. dd, 1H, H5A, J_5A,6_ = 8.3, J_5,5_ = 14.2), 4.55 (ddd, 1H, H7A, J_7A,6A_ = 5.1, J_7A,6B_ = 12.7, J_7,7_ = 17.9), 5.12 (d, 1H, H11, J_11,12_ = 1.7), 5.47 (s, 1H, H13B), 6.06 (dd, 1H, H2, J_2,3_ = 2.2, J_1,2_ = 3.4), 6.17 (dd, 1H, H1, J_1,3_ = 1.7, J_1,2_ = 3.4), 6.40 (dd, 1H, H12, J_11,12_ = 1.7, J_12,13_ = 5.8), 6.55 (dd, 1H, H3, J_1,3_ = 1.7, J_2,3_ = 2.2), 6.59 (d, 1H, H13, J_12,13_ = 5.8). EI—MS (70 eV) *m/z* (*rel*. intensity): 300 [*M*]^+^ (84), 282 (5), 271 (15), 254 (73), 237 (20), 225 (66), 211 (12), 202 (100), 187 (26), 172 (19), 158 (6), 144 (6), 135 (31), 106 (33), 98 (30), 91 (23), 79 (65), 54 (60), 43 (55). Anal. Calcd for C_17_H_18_N_2_O_4_: C, 64.96; H, 5.77; N, 8.91. Found: C, 64.88; H, 5.53; N, 8.66.
Geometry. All e.s.d.'s (except the e.s.d. in the dihedral angle between two l.s. planes) are estimated using the full covariance matrix. The cell e.s.d.'s are taken into account individually in the estimation of e.s.d.'s in distances, angles and torsion angles; correlations between e.s.d.'s in cell parameters are only used when they are defined by crystal symmetry. An approximate (isotropic) treatment of cell e.s.d.'s is used for estimating e.s.d.'s involving l.s. planes.
Refinement. Refinement of *F*^2^ against ALL reflections. The weighted *R*-factor *wR* and goodness of fit *S* are based on *F*^2^, conventional *R*-factors *R* are based on *F*, with *F* set to zero for negative *F*^2^. The threshold expression of *F*^2^ > σ(*F*^2^) is used only for calculating *R*-factors(gt) *etc*. and is not relevant to the choice of reflections for refinement. *R*-factors based on *F*^2^ are statistically about twice as large as those based on *F*, and *R*- factors based on ALL data will be even larger.

### Fractional atomic coordinates and isotropic or equivalent isotropic displacement parameters (Å^2^)

**Table:**

	*x*	*y*	*z*	*U*_iso_*/*U*_eq_	
O1	0.21252 (7)	0.13743 (9)	0.19684 (5)	0.01893 (16)	
O2	0.12840 (7)	0.15880 (8)	−0.04077 (6)	0.01879 (15)	
O3	0.00054 (7)	0.29911 (8)	0.00561 (6)	0.01676 (15)	
C1	0.72407 (9)	0.21971 (11)	0.19832 (7)	0.01507 (18)	
H1	0.7658	0.3084	0.2280	0.018*	
C2	0.77184 (9)	0.10722 (12)	0.15513 (7)	0.01717 (19)	
H2	0.8510	0.1073	0.1498	0.021*	
C3	0.68230 (9)	−0.00134 (11)	0.12259 (7)	0.01630 (19)	
H3	0.6892	−0.0906	0.0911	0.020*	
N4	0.58103 (8)	0.04029 (9)	0.14314 (6)	0.01360 (16)	
C5	0.46763 (9)	−0.04890 (11)	0.12104 (7)	0.01633 (18)	
H5A	0.4717	−0.1360	0.0818	0.020*	
H5B	0.3940	0.0110	0.0811	0.020*	
C6	0.44989 (9)	−0.10258 (11)	0.21236 (7)	0.01676 (19)	
H6A	0.5128	−0.1802	0.2446	0.020*	
H6B	0.3658	−0.1479	0.1930	0.020*	
C7	0.46293 (9)	0.02321 (11)	0.28511 (7)	0.01455 (18)	
H7A	0.4117	−0.0004	0.3233	0.017*	
H7B	0.5514	0.0302	0.3312	0.017*	
N8	0.42359 (7)	0.16558 (9)	0.23701 (6)	0.01275 (15)	
C9	0.30159 (9)	0.20771 (10)	0.19421 (7)	0.01331 (17)	
C9A	0.29876 (8)	0.35968 (10)	0.14869 (7)	0.01202 (17)	
H9A	0.2956	0.4404	0.1939	0.014*	
C10	0.20435 (8)	0.39060 (10)	0.04251 (7)	0.01249 (17)	
H10	0.1606	0.4872	0.0393	0.015*	
C11	0.29855 (8)	0.40367 (10)	−0.00989 (7)	0.01305 (17)	
H11	0.2602	0.3913	−0.0824	0.016*	
C12	0.37240 (9)	0.54653 (11)	0.02527 (7)	0.01537 (18)	
H12	0.3626	0.6368	−0.0099	0.018*	
C13	0.45390 (9)	0.51866 (10)	0.11585 (7)	0.01456 (18)	
H13	0.5158	0.5835	0.1582	0.017*	
C13A	0.42597 (8)	0.36124 (10)	0.13604 (6)	0.01148 (17)	
C13B	0.51312 (8)	0.26159 (10)	0.21615 (6)	0.01180 (17)	
H13B	0.5593	0.3234	0.2750	0.014*	
C13C	0.60588 (8)	0.17628 (10)	0.18918 (6)	0.01212 (17)	
O14	0.38943 (6)	0.29270 (7)	0.04125 (5)	0.01203 (14)	
C14	0.11017 (8)	0.26807 (10)	−0.00121 (7)	0.01313 (17)	
C15	−0.09493 (10)	0.18619 (12)	−0.03298 (9)	0.0222 (2)	
H15A	−0.1745	0.2235	−0.0332	0.033*	
H15B	−0.0700	0.0972	0.0079	0.033*	
H15C	−0.1049	0.1615	−0.0998	0.033*	

### Atomic displacement parameters (Å^2^)

**Table:**

	*U*^11^	*U*^22^	*U*^33^	*U*^12^	*U*^13^	*U*^23^
O1	0.0149 (3)	0.0210 (4)	0.0219 (3)	−0.0005 (3)	0.0083 (3)	0.0055 (3)
O2	0.0196 (3)	0.0164 (3)	0.0230 (4)	−0.0020 (3)	0.0111 (3)	−0.0050 (3)
O3	0.0122 (3)	0.0144 (3)	0.0241 (3)	0.0005 (2)	0.0075 (3)	0.0001 (3)
C1	0.0129 (4)	0.0176 (4)	0.0145 (4)	0.0001 (3)	0.0051 (3)	−0.0006 (3)
C2	0.0154 (4)	0.0220 (5)	0.0155 (4)	0.0049 (4)	0.0074 (3)	0.0020 (4)
C3	0.0209 (5)	0.0159 (4)	0.0138 (4)	0.0062 (4)	0.0086 (3)	0.0015 (3)
N4	0.0158 (4)	0.0116 (4)	0.0135 (3)	0.0007 (3)	0.0058 (3)	−0.0009 (3)
C5	0.0183 (4)	0.0132 (4)	0.0156 (4)	−0.0027 (3)	0.0045 (3)	−0.0025 (3)
C6	0.0168 (4)	0.0127 (4)	0.0199 (4)	−0.0014 (3)	0.0060 (4)	0.0012 (3)
C7	0.0144 (4)	0.0142 (4)	0.0139 (4)	0.0012 (3)	0.0043 (3)	0.0036 (3)
N8	0.0122 (3)	0.0126 (3)	0.0141 (3)	0.0007 (3)	0.0058 (3)	0.0022 (3)
C9	0.0144 (4)	0.0145 (4)	0.0117 (4)	0.0013 (3)	0.0058 (3)	0.0003 (3)
C9A	0.0122 (4)	0.0120 (4)	0.0124 (4)	0.0016 (3)	0.0054 (3)	0.0001 (3)
C10	0.0123 (4)	0.0113 (4)	0.0138 (4)	0.0015 (3)	0.0049 (3)	0.0002 (3)
C11	0.0133 (4)	0.0125 (4)	0.0136 (4)	0.0016 (3)	0.0054 (3)	0.0013 (3)
C12	0.0169 (4)	0.0118 (4)	0.0192 (4)	0.0003 (3)	0.0090 (3)	0.0021 (3)
C13	0.0160 (4)	0.0107 (4)	0.0187 (4)	−0.0010 (3)	0.0085 (3)	−0.0008 (3)
C13A	0.0125 (4)	0.0106 (4)	0.0119 (4)	0.0001 (3)	0.0053 (3)	−0.0015 (3)
C13B	0.0113 (4)	0.0119 (4)	0.0120 (4)	−0.0003 (3)	0.0044 (3)	−0.0010 (3)
C13C	0.0127 (4)	0.0119 (4)	0.0114 (4)	0.0009 (3)	0.0042 (3)	−0.0004 (3)
O14	0.0134 (3)	0.0115 (3)	0.0109 (3)	0.0019 (2)	0.0044 (2)	−0.0006 (2)
C14	0.0126 (4)	0.0140 (4)	0.0125 (4)	0.0017 (3)	0.0047 (3)	0.0024 (3)
C15	0.0159 (4)	0.0197 (5)	0.0320 (5)	−0.0038 (4)	0.0105 (4)	−0.0008 (4)

### Geometric parameters (Å, °)

**Table:**

O1—C9	1.2241 (12)	N8—C13B	1.4728 (12)
O2—C14	1.2069 (12)	C9—C9A	1.5227 (13)
O3—C14	1.3417 (11)	C9A—C13A	1.5557 (13)
O3—C15	1.4482 (12)	C9A—C10	1.5584 (13)
C1—C13C	1.3790 (13)	C9A—H9A	1.0000
C1—C2	1.4217 (13)	C10—C14	1.5113 (13)
C1—H1	0.9500	C10—C11	1.5709 (13)
C2—C3	1.3709 (15)	C10—H10	1.0000
C2—H2	0.9500	C11—O14	1.4379 (11)
C3—N4	1.3735 (12)	C11—C12	1.5224 (13)
C3—H3	0.9500	C11—H11	1.0000
N4—C13C	1.3790 (12)	C12—C13	1.3355 (13)
N4—C5	1.4648 (12)	C12—H12	0.9500
C5—C6	1.5276 (14)	C13—C13A	1.5112 (13)
C5—H5A	0.9900	C13—H13	0.9500
C5—H5B	0.9900	C13A—O14	1.4437 (11)
C6—C7	1.5335 (14)	C13A—C13B	1.5211 (12)
C6—H6A	0.9900	C13B—C13C	1.4964 (12)
C6—H6B	0.9900	C13B—H13B	1.0000
C7—N8	1.4540 (12)	C15—H15A	0.9800
C7—H7A	0.9900	C15—H15B	0.9800
C7—H7B	0.9900	C15—H15C	0.9800
N8—C9	1.3602 (12)		
			
C14—O3—C15	115.05 (8)	C14—C10—C9A	114.31 (7)
C13C—C1—C2	107.32 (9)	C14—C10—C11	111.46 (7)
C13C—C1—H1	126.3	C9A—C10—C11	99.53 (7)
C2—C1—H1	126.3	C14—C10—H10	110.4
C3—C2—C1	107.17 (9)	C9A—C10—H10	110.4
C3—C2—H2	126.4	C11—C10—H10	110.4
C1—C2—H2	126.4	O14—C11—C12	102.00 (7)
C2—C3—N4	108.73 (8)	O14—C11—C10	101.10 (7)
C2—C3—H3	125.6	C12—C11—C10	107.40 (7)
N4—C3—H3	125.6	O14—C11—H11	114.9
C3—N4—C13C	108.72 (8)	C12—C11—H11	114.9
C3—N4—C5	124.67 (8)	C10—C11—H11	114.9
C13C—N4—C5	126.59 (8)	C13—C12—C11	105.73 (8)
N4—C5—C6	113.07 (8)	C13—C12—H12	127.1
N4—C5—H5A	109.0	C11—C12—H12	127.1
C6—C5—H5A	109.0	C12—C13—C13A	104.76 (8)
N4—C5—H5B	109.0	C12—C13—H13	127.6
C6—C5—H5B	109.0	C13A—C13—H13	127.6
H5A—C5—H5B	107.8	O14—C13A—C13	102.28 (7)
C5—C6—C7	112.43 (8)	O14—C13A—C13B	111.49 (7)
C5—C6—H6A	109.1	C13—C13A—C13B	125.58 (8)
C7—C6—H6A	109.1	O14—C13A—C9A	100.37 (7)
C5—C6—H6B	109.1	C13—C13A—C9A	108.53 (7)
C7—C6—H6B	109.1	C13B—C13A—C9A	105.84 (7)
H6A—C6—H6B	107.8	N8—C13B—C13C	113.03 (8)
N8—C7—C6	112.31 (8)	N8—C13B—C13A	101.75 (7)
N8—C7—H7A	109.1	C13C—C13B—C13A	114.94 (7)
C6—C7—H7A	109.1	N8—C13B—H13B	108.9
N8—C7—H7B	109.1	C13C—C13B—H13B	108.9
C6—C7—H7B	109.1	C13A—C13B—H13B	108.9
H7A—C7—H7B	107.9	N4—C13C—C1	108.06 (8)
C9—N8—C7	123.21 (8)	N4—C13C—C13B	123.79 (8)
C9—N8—C13B	115.22 (8)	C1—C13C—C13B	128.00 (9)
C7—N8—C13B	120.66 (7)	C11—O14—C13A	95.53 (6)
O1—C9—N8	125.20 (9)	O2—C14—O3	123.77 (9)
O1—C9—C9A	127.32 (9)	O2—C14—C10	124.95 (9)
N8—C9—C9A	107.40 (8)	O3—C14—C10	111.22 (8)
C9—C9A—C13A	101.72 (7)	O3—C15—H15A	109.5
C9—C9A—C10	119.79 (8)	O3—C15—H15B	109.5
C13A—C9A—C10	101.68 (7)	H15A—C15—H15B	109.5
C9—C9A—H9A	110.9	O3—C15—H15C	109.5
C13A—C9A—H9A	110.9	H15A—C15—H15C	109.5
C10—C9A—H9A	110.9	H15B—C15—H15C	109.5
			
C13C—C1—C2—C3	−0.86 (11)	C10—C9A—C13A—C13	70.29 (8)
C1—C2—C3—N4	0.56 (11)	C9—C9A—C13A—C13B	−28.44 (9)
C2—C3—N4—C13C	−0.05 (10)	C10—C9A—C13A—C13B	−152.58 (7)
C2—C3—N4—C5	−178.73 (8)	C9—N8—C13B—C13C	−133.68 (8)
C3—N4—C5—C6	114.00 (10)	C7—N8—C13B—C13C	35.75 (11)
C13C—N4—C5—C6	−64.44 (12)	C9—N8—C13B—C13A	−9.87 (10)
N4—C5—C6—C7	49.81 (11)	C7—N8—C13B—C13A	159.56 (8)
C5—C6—C7—N8	30.48 (11)	O14—C13A—C13B—N8	−84.63 (8)
C6—C7—N8—C9	80.15 (11)	C13—C13A—C13B—N8	151.13 (8)
C6—C7—N8—C13B	−88.42 (10)	C9A—C13A—C13B—N8	23.60 (9)
C7—N8—C9—O1	5.44 (15)	O14—C13A—C13B—C13C	37.88 (10)
C13B—N8—C9—O1	174.58 (9)	C13—C13A—C13B—C13C	−86.36 (11)
C7—N8—C9—C9A	−177.68 (8)	C9A—C13A—C13B—C13C	146.11 (8)
C13B—N8—C9—C9A	−8.54 (10)	C3—N4—C13C—C1	−0.50 (10)
O1—C9—C9A—C13A	−160.63 (9)	C5—N4—C13C—C1	178.15 (9)
N8—C9—C9A—C13A	22.59 (9)	C3—N4—C13C—C13B	175.47 (8)
O1—C9—C9A—C10	−49.71 (13)	C5—N4—C13C—C13B	−5.89 (14)
N8—C9—C9A—C10	133.51 (8)	C2—C1—C13C—N4	0.83 (10)
C9—C9A—C10—C14	7.94 (11)	C2—C1—C13C—C13B	−174.91 (9)
C13A—C9A—C10—C14	118.89 (8)	N8—C13B—C13C—N4	29.70 (12)
C9—C9A—C10—C11	−110.93 (9)	C13A—C13B—C13C—N4	−86.51 (11)
C13A—C9A—C10—C11	0.01 (8)	N8—C13B—C13C—C1	−155.17 (9)
C14—C10—C11—O14	−84.18 (8)	C13A—C13B—C13C—C1	88.61 (11)
C9A—C10—C11—O14	36.79 (8)	C12—C11—O14—C13A	49.61 (8)
C14—C10—C11—C12	169.35 (7)	C10—C11—O14—C13A	−61.07 (7)
C9A—C10—C11—C12	−69.68 (8)	C13—C13A—O14—C11	−51.18 (8)
O14—C11—C12—C13	−30.93 (9)	C13B—C13A—O14—C11	172.30 (7)
C10—C11—C12—C13	74.90 (9)	C9A—C13A—O14—C11	60.57 (7)
C11—C12—C13—C13A	−1.82 (10)	C15—O3—C14—O2	3.79 (14)
C12—C13—C13A—O14	33.99 (9)	C15—O3—C14—C10	−178.93 (8)
C12—C13—C13A—C13B	162.06 (9)	C9A—C10—C14—O2	−84.24 (11)
C12—C13—C13A—C9A	−71.52 (9)	C11—C10—C14—O2	27.65 (13)
C9—C9A—C13A—O14	87.60 (7)	C9A—C10—C14—O3	98.52 (9)
C10—C9A—C13A—O14	−36.53 (8)	C11—C10—C14—O3	−149.60 (8)
C9—C9A—C13A—C13	−165.57 (7)		

### Hydrogen-bond geometry (Å, °)

**Table:**

*D*—H···*A*	*D*—H	H···*A*	*D*···*A*	*D*—H···*A*
C9A—H9A···O1^i^	1.00	2.43	3.4334 (13)	180

Symmetry codes: (i) −*x*+1/2, *y*+1/2, −*z*+1/2.

## References

[bb1] Bruker (2001). *SAINT-Plus* Bruker AXS Inc., Madison, Wisconsin, USA.

[bb2] Bruker (2005). *APEX2* Bruker AXS Inc., Madison, Wisconsin, USA.

[bb3] Donohoe, T. J. (2000). *Science of Synthesis*, edited by E. J. Thomas, Vol. 10, p. 653. Stuttgart: Georg Thieme Verlag.

[bb4] Jones, G. B. & Chapman, B. J. (1996). *Comprehensive Heterocyclic Chemistry II*, edited by A. R. Katrizky, C. W. Rees & E. F. V. Scriven, Vol. 2, p. 1. Oxford: Pergamon.

[bb5] Sheldrick, G. M. (2003). *SADABS* Bruker AXS Inc., Madison, Wisconsin, USA.

[bb6] Sheldrick, G. M. (2008). *Acta Cryst.* A**64**, 112–122. 10.1107/S0108767307043930 18156677

[bb7] Vogel, P., Cossy, J., Plumet, J. & Arjona, O. (1999). *Tetrahedron*, **55**, 13521–13642.

[bb8] Zubkov, F. I., Ershova, J. D., Orlova, A. A., Zaytsev, V. P., Nikitina, E. V., Peregudov, A. S., Gurbanov, A. V., Borisov, R. S., Khrustalev, V. N., Maharramov, A. M. & Varlamov, A. V. (2009). *Tetrahedron*, **65**, 3789–3803.

[bb9] Zubkov, F. I., Galeev, T. R., Nikitina, E. V., Lazenkova, I. V., Zaytsev, V. P. & Varlamov, A. V. (2010). *Synlett*, pp. 2063–2066.

[bb10] Zubkov, F. I., Nikitina, E. V. & Varlamov, A. V. (2005). *Russ. Chem. Rev.* **74**, 639–669.

[bb11] Zubkov, F. I., Zaytsev, V. P., Nikitina, E. V., Khrustalev, V. N., Gozun, S. V., Boltukhina, E. V. & Varlamov, A. V. (2011). *Tetrahedron*, **67** In the press, doi: 10.1016/j.tet.2011.09.099.

